# Oncological Outcomes of Breast-Conserving Surgery Versus Mastectomy in Invasive Lobular Breast Cancer: A Single-Center Retrospective Study

**DOI:** 10.3390/medicina62040645

**Published:** 2026-03-28

**Authors:** Simay Çokgezer, Aysel Safaraliyeva, Sevde Topuz, Muhammet Şenkal, Naziye Ak, Didem Taştekin, Pınar Mualla Saip

**Affiliations:** 1 Department of Medical Oncology, Institute of Oncology, Istanbul University, 34093 Istanbul, Turkey; aknaziyeak@gmail.com (N.A.); didem_doktor@hotmail.com (D.T.); pinarsaip@gmail.com (P.M.S.); 2 Department of Internal Medicine, Istanbul Faculty of Medicine, Istanbul University, 34093 Istanbul, Turkey; ayselsafaraliyeva19@gmail.com (A.S.); azrasevdet@gmail.com (S.T.); mamulli.muhammetsenkal@gmail.com (M.Ş.)

**Keywords:** overall survival, invasive lobular carcinoma, mastectomy, breast conserving surgery, progression-free survival

## Abstract

*Background and Objectives*: Invasive lobular carcinoma (ILC) is a breast cancer subtype with a controversial surgical management due to its diffuse infiltrative growth pattern and increased tendency for multicentricity. This study aimed to compare the effects of breast-conserving surgery (BCS) and mastectomy on long-term overall survival (OS) and progression-free survival (PFS) in patients diagnosed with ILC. *Materials and Methods*: In this single-center, retrospective, observational study, 255 patients with histopathologically confirmed ILC between 2017 and 2025 were included. Patients who underwent surgical treatment were divided into two groups according to the surgical approach: BCS (*n* = 94) and mastectomy (*n* = 141). Survival analyses were performed using the Kaplan–Meier method, and comparisons between groups were assessed with the log-rank test. Factors affecting survival were evaluated using Cox regression analysis. *Results*: The median age of the patients was 53 years (range, 28–85), and the median follow-up duration was 31.8 months. Of the cases, 76.9% were classic-type ILC and 70.9% had stage I–II disease. The rate of negative surgical margins was 87.6%. No statistically significant differences were observed between the BCS and mastectomy groups in terms of estimated median PFS (87.4 months vs. 86.7 months; *p* > 0.05) or estimated median OS (87.7 months vs. 115.7 months; *p* > 0.05). Multivariable analyses demonstrated that the type of surgery was not an independent prognostic factor for survival. *Conclusions*: This study shows that, with appropriate patient selection and adequate surgical margin control, BCS provides oncologic survival outcomes comparable to mastectomy in ILC. The choice of surgical approach should be individualized based on tumor biology, stage, and multidisciplinary evaluation rather than histological subtype alone.

## 1. Introduction

Invasive lobular carcinoma (ILC) accounts for approximately 15% of all breast cancers and is the second most common histological subtype of invasive breast carcinoma. Owing to its distinctive histopathological, molecular, and biological characteristics, ILC is currently recognized as a separate clinical and biological entity, distinct from invasive breast carcinoma of no special type [1,2]. Patients with ILC are typically diagnosed at an older age, and the tumors are frequently hormone receptor-positive with low rates of HER2 positivity. However, despite a lower histological grade and slower proliferation rate, the higher incidence of multifocality, multicentricity, and bilaterality makes the clinical management of ILC more complex [3,4,5,6]. The biological basis of this clinical behavior lies in the loss of E-cadherin (CDH1) function, a hallmark feature of ILC. This alteration disrupts intercellular adhesion and leads to a non-cohesive, single-file infiltrative growth pattern of tumor cells [7]. The coexistence of these clinical and pathological features underscores the need for treatment strategies in ILC to be tailored according to histological subtype [8].

The diffuse infiltrative growth pattern commonly observed in ILC reduces the contrast between the tumor and surrounding tissues, making the clinical and radiological boundaries difficult to delineate and complicating accurate assessment of local disease extent [9]. The literature indicates that the sensitivity of mammography for detecting ILC is significantly lower than that for invasive ductal carcinoma (IDC) (34% vs. 81%, respectively), and this rate may decrease to as low as 11% in patients with dense breast tissue [10]. This limited diagnostic performance often results in underestimation of tumor size on imaging compared with pathological measurements and may hinder the achievement of negative surgical margins, thereby making the choice of surgical approach controversial [9]. In this context, magnetic resonance imaging (MRI) plays a critical role in surgical planning, with a reported sensitivity of up to 93% and an improved ability to detect additional tumor foci. Although the use of MRI has been shown to reduce re-excision rates, its impact on long-term oncologic outcomes remains controversial due to its lower specificity and potential association with increased mastectomy rates [11]. Therefore, imaging strategies and the selection of surgical approaches in ILC should be individualized, taking into account both their potential benefits and limitations.

The oncologic safety of breast conserving surgery (BCS) in the surgical management of ILC has long been debated because of the tumor’s diffuse growth pattern and high rates of multicentricity [12]. However, widely accepted evidence in the literature indicates that, with appropriate patient selection, BCS provides outcomes equivalent to mastectomy in terms of local control and survival [3,13]. Nevertheless, one of the most significant clinical challenges after BCS in ILC is the higher rate of reoperation (re-excision) due to positive surgical margins compared with invasive ductal carcinoma (IDC) [14,15]. Although some studies have suggested that BCS may offer a survival advantage over mastectomy, particularly in stage II or higher disease, the ultimate impact of surgical type on survival is largely influenced by patient-specific factors and tumor biology [16].

In light of these data, the distinctive biological and clinical features of ILC necessitate considerations beyond standard approaches in the surgical decision-making process. Given the ongoing debate in the literature regarding surgical strategies and the heterogeneity of reported outcomes, analysis of long-term clinical data from our institution is of particular importance. The primary aim of this study was to compare the oncologic safety of BCS and mastectomy in our cohort of patients who underwent surgical treatment for ILC, using overall survival (OS) and progression-free survival (PFS) as endpoints. By evaluating the long-term survival outcomes associated with different surgical approaches, this study seeks to contribute to the clinical decision-making process regarding surgical management in ILC.

## 2. Materials and Methods

### 2.1. Study Design and Patient Selection

This was a single-center, retrospective, observational study in which patients with a diagnosis of ILC were evaluated. Patients who were followed in our clinic and had a histopathologically confirmed diagnosis of ILC were included in the study. Demographic data, clinical features, tumor characteristics, and information on surgical and adjuvant treatments were obtained from patient files and electronic record systems.

Patients with accessible clinical and pathological data at the time of diagnosis were included in the study, whereas those with missing clinical, pathological, or follow-up data were excluded from the analysis.

For the description of demographic and clinical characteristics, all patients diagnosed with stage I–IV ILC at presentation were evaluated. Survival comparisons among patients who underwent surgical treatment were limited to cases with stage I–III disease at diagnosis who received curative-intent surgery.

### 2.2. Clinical and Pathological Evaluation

Patients’ demographic and clinical characteristics (age at diagnosis, menopausal status, body mass index, presence of comorbidities, ECOG performance status, and family history) were recorded. Based on pathology reports, tumor size, histological subtype and grade, estrogen and progesterone receptor status, HER2 status, Ki-67 proliferation index (%), presence of lymphovascular invasion, and pathological stage were evaluated.

Hormone receptor status [estrogen receptor (ER) and progesterone receptor (PR)] was assessed using the immunohistochemical (IHC) method; cases with nuclear staining in at least 1% of tumor cells were considered hormone receptor-positive. HER2 status was evaluated using IHC staining and, when necessary, the silver in situ hybridization (SISH) method. In IHC scoring, cases with scores of 0 and 1+ were considered HER2-negative. For IHC 2+ cases, SISH testing was performed; 2+ cases that were SISH-negative were classified as HER2-negative, and those that were SISH-positive were classified as HER2-positive. All cases with IHC 3+ were considered HER2-positive. Surgical margin status was evaluated according to the “no ink on tumor” principle.

Preoperative imaging evaluation included mammography and breast ultrasonography in all patients. In addition, breast MRI was performed in the majority of patients (approximately 90%) to better assess tumor extent and detect possible multifocal or multicentric disease, which may influence surgical planning in patients with invasive lobular carcinoma.

In patients who underwent surgical treatment, the type of surgery was classified as BCS or mastectomy. The choice of surgical approach was determined by a multidisciplinary team based on tumor characteristics, disease extent, and patient preference. BCS was generally considered appropriate for patients with localized tumors in whom complete tumor excision with an acceptable cosmetic outcome was feasible. Mastectomy was preferred in cases of large tumor size relative to breast volume, multifocal or multicentric disease, contraindications to radiotherapy, or patient preference. Surgical margin status was assessed according to standard pathological evaluation. A negative margin was defined as the absence of tumor cells at the inked margin. In cases with positive surgical margins, re-excision was performed when feasible to achieve clear margins. Axillary surgical approaches were recorded as limited axillary surgery and axillary lymph node dissection (ALND). Limited axillary surgery included sentinel lymph node biopsy (SLNB), low-level axillary curettage, non-sentinel lymph node removal (non-SLN), and axillary sampling procedures. In patients who received neoadjuvant treatment, residual tumor burden and the rate of regressive fibrosis were also recorded.

### 2.3. Follow-Up and Endpoints

Patients were followed with regular clinical and radiological evaluations from the date of diagnosis. PFS was defined as the time from the date of diagnosis to disease progression, recurrence, or the development of distant metastasis. OS was calculated as the time from the date of diagnosis to death from any cause. Survival times were calculated from the date of diagnosis to provide a common starting point for the entire cohort, particularly given that a proportion of patients received neoadjuvant treatment prior to surgery. Using the date of diagnosis as the starting point allowed consistent inclusion of both patients undergoing upfront surgery and those receiving neoadjuvant therapy. Patients without progression or death during follow-up were censored at the date of their last visit.

### 2.4. Statistical Analysis

Statistical analyses were performed using SPSS software (IBM SPSS Statistics, version 28.0; IBM Corp., Armonk, NY, USA). For continuous variables, mean ± standard deviation, median, minimum, and maximum values were reported; for categorical variables, frequencies and percentages were used. The distribution of variables was assessed using the Kolmogorov–Smirnov and Shapiro–Wilk tests.

Kaplan–Meier analysis was used for survival analyses, and comparisons between groups were performed using the log-rank test. Cox regression analysis was applied to evaluate factors that might affect progression-free and overall survival. Statistical significance was defined as *p* < 0.05.

### 2.5. Ethical Approval

This study was approved by the Clinical Research Ethics Committee of Istanbul University, Istanbul Faculty of Medicine (Date: 10 February 2026, No: 3904607). The study was conducted in accordance with the principles of the Declaration of Helsinki, and the requirement for informed consent was waived by the ethics committee due to the retrospective design.

## 3. Results

### 3.1. Patient Population and Clinical Characteristics

A total of 255 patients with a diagnosis of ILC were included in the study. The median age at diagnosis was 53 years (range: 28–85), and 58% of the patients (*n* = 148) were in the ≥50-year age group. Postmenopausal patients accounted for 56.5% (*n* = 144), and 98.5% of the patients (*n* = 251) had an ECOG performance status of 0–1.

Comorbid conditions were identified in 119 patients (46.6%), whereas 136 patients (53.3%) had no documented comorbidity. The most frequently observed comorbidities were hypertension (n = 46, 18.0%), diabetes mellitus (*n* = 23, 9%), thyroid disorders (*n* = 14, 5.5%), hyperlipidemia (*n* = 12, 4.7%), and respiratory diseases including asthma or chronic obstructive pulmonary disease (*n* = 11, 4.3%). Cardiovascular diseases such as coronary artery disease were observed in 8 patients (3.1%), while chronic kidney disease was present in 4 patients (1.5%). Other less common comorbid conditions included autoimmune diseases, neurological disorders, anemia, migraine, nephrolithiasis, and psychiatric disorders. In addition, 22.0% of patients (*n* = 56) had a body mass index (BMI) ≥ 30. Regarding family history, 24.3% of patients (*n* = 62) had a first-degree relative with a history of breast cancer. BRCA1 or BRCA2 positivity was detected in 3.2% of patients (*n* = 8), whereas BRCA status was unknown in the majority of patients (85.1%, *n* = 217). Demographic and clinical characteristics of the patients are presented in detail in Table 1.

### 3.2. Tumor and Pathological Characteristics

The median tumor size at diagnosis was 19 mm (range: 5–95). Regarding histological subtype distribution, 76.9% of cases were classified as classic-type ILC, while the remainder were pleomorphic or other variants. The vast majority of tumors were ER-positive (91.4%), PR-positive (81.9%), and HER2-negative (93.3%). The proportion of patients with a Ki-67 proliferation index < 20% was 59.2% (*n* = 151). Lymphovascular invasion was detected in 30.6% of cases (*n* = 78), and multicentricity was observed in 20.8%. In terms of pathological staging, most patients were in the stage I–II group (70.9%). Tumor and pathological characteristics are summarized in Table 2.

### 3.3. Treatment Characteristics

Preoperative breast MRI was performed in approximately 90% of patients and was used to evaluate tumor extent and potential multifocal or multicentric disease during surgical planning. Surgical treatment was performed in 92.2% of the patients (*n* = 235), while 7.8% (*n* = 20) were followed without surgery [due to reasons such as metastatic disease, inoperability, or patient refusal]. Based on the 235 patients who underwent surgery, 40% (*n* = 94) received BCS and 60% (*n* = 141) underwent mastectomy. In terms of axillary surgical approach, 75.7% of cases underwent SLNB or limited axillary surgery, while 15.7% underwent ALND. Negative surgical margins were achieved in 87.6% of cases. In patients who received neoadjuvant treatment, residual tumor burden, the presence of regressive fibrosis, and necrosis were additionally recorded. Regarding adjuvant treatment, the majority of patients received endocrine therapy, and adjuvant chemotherapy and/or radiotherapy were administered in cases with appropriate clinical and pathological features. Details of the surgical approach, type of axillary surgery, and neoadjuvant and adjuvant treatments are presented in Table 3. The baseline clinicopathological characteristics of patients according to surgical approach are presented in Table 4. Notably, patients undergoing mastectomy had more advanced disease characteristics, including larger tumor size, higher nodal involvement, increased rates of multicentric disease, and more frequent use of neoadjuvant chemotherapy.

### 3.4. Progression Free Survival Analysis

**Figure 1:**
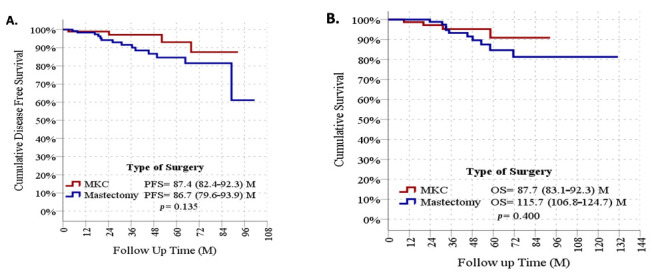
Survival analyses according to surgical approach. (**A**) Kaplan–Meier curves for progression-free survival according to surgical approach. (**B**) Kaplan–Meier curves for overall survival according to surgical approach.

The median follow-up duration was 31.8 months. The estimated PFS was 87.4 months in patients who underwent BCS and 86.7 months in those who underwent mastectomy. Comparative analysis showed no statistically significant difference in PFS between the two surgical approaches. Kaplan–Meier survival curves are shown in Figure 1A, and the comparative analysis results are presented in Table 5.

### 3.5. Overall Survival Analysis

The estimated OS was 87.7 months in patients who underwent BCS and 115.7 months in those who underwent mastectomy. No statistically significant difference in OS was observed between the BCS and mastectomy groups (*p* > 0.05). Kaplan–Meier curves for OS are shown in Figure 1B, and the comparative analysis results are presented in Table 6.

### 3.6. Subgroup Analyses

In the subgroup analyses, the effect of surgical type on survival was evaluated separately according to clinicopathological variables such as age, tumor size, pathologic stage, nodal involvement, multifocal/multicentric disease, neoadjuvant chemotherapy, adjuvant chemotherapy, Ki-67 proliferation index, and surgical margin status. In Cox regression analyses, no significant effect of surgical type on overall survival was observed in the <50 years and ≥50 years subgroups (*p* > 0.05 for both groups). Similarly, no statistically significant difference in overall survival was observed according to surgical type across subgroups defined by tumor size (≤20 mm vs. >20 mm), pathologic stage, nodal status, presence of multifocal/multicentric disease, neoadjuvant chemotherapy, adjuvant chemotherapy, Ki-67 proliferation index, and surgical margin status (all *p* > 0.05).

Overall, these findings consistently demonstrated that the type of surgery was not associated with survival outcomes across all evaluated subgroups (Table 7).

## 4. Discussion

ILC continues to be a challenging entity for clinicians in surgical management due to its characteristic diffuse infiltrative growth pattern and the limitations of diagnostic imaging. In particular, the inability to clearly define tumor margins in the preoperative period and the high risk of multicentricity have long supported the perception that mastectomy is a safer option. However, the results of the present study support that, when patients are carefully selected based on tumor size, disease extent, and the feasibility of achieving complete tumor excision with acceptable cosmetic outcomes, BCS represents an oncologically safe option in patients with ILC. In our cohort of 255 patients, no statistically significant difference was observed in either OS or PFS between patients who underwent BCS and those who underwent mastectomy after a median follow-up of 31.8 months. These findings suggest that BCS can provide oncologically comparable outcomes to mastectomy, provided that adequate surgical margins are achieved, consistent with the “no tumor on ink” principle, and highlight that the type of surgery itself does not appear to be an independent determinant of survival in appropriately selected patients.

The primary difficulty in the clinical management of ILC arises from its distinctive histomorphological structure, characterized by the loss of function of CDH1, an intercellular adhesion molecule, resulting in a non-cohesive growth pattern with tumor cells infiltrating the stroma in single-file arrangements [17]. This diffuse infiltrative growth pattern prevents the formation of a prominent desmoplastic reaction, leading to low-contrast presentation on both physical examination and conventional mammography, and consequently to underestimation of lesion size compared with pathological boundaries [18]. When these diagnostic limitations are combined with the frequent multifocal and multicentric growth pattern observed in ILC, surgical margin management becomes more complex; this results in significantly higher re-excision and final mastectomy rates compared with IDC [3]. In this context, MRI plays a critical role in detecting additional tumor foci due to its superior sensitivity compared with conventional methods; however, because of its lower specificity, it has the potential to increase mastectomy rates without providing a proven survival advantage [19]. Indeed, data from national screening programs emphasize that, in ILC, the primary factor determining the type of surgery is not the biological behavior of the tumor, but rather the uncertainty in accurately assessing the true extent of disease in the preoperative period [3]. Consequently, the selection of the surgical approach in ILC represents a complex clinical decision-making process that should not rely solely on imaging findings, but should instead incorporate tumor biology, radiological extent, the feasibility of achieving negative surgical margins, and multidisciplinary evaluation [20].

In ILC, the oncologic safety of BCS has long been a matter of debate due to the infiltrative growth pattern and high rates of positive surgical margins; however, contemporary series have shown that when negative surgical margins are achieved, BCS provides survival and local control outcomes comparable to mastectomy [21]. In large institutional series, when evaluated according to surgical type, no significant differences were observed between ILC patients treated with BCS and those treated with mastectomy in terms of local recurrence rates, disease-free survival, or OS; the main determinants of survival were stage and hormone receptor status rather than histology [4]. In addition, long-term follow-up data support that, despite the increased rates of multicentricity and bilaterality in ILC, BCS performed with appropriate patient selection is safe in terms of local control, and mastectomy is not routinely a superior strategy [4,22]. These findings indicate that the high re-excision rates in ILC are more closely related to tumor biology and imaging limitations than to inadequacy of the surgical approach; when surgical margin management is optimized, BCS represents an oncologically equivalent option to mastectomy [21,23]. More recently, large contemporary cohorts have also suggested that breast-conserving surgery may be associated with favorable long-term oncologic outcomes. In a retrospective analysis including 607 breast cancer patients treated with neoadjuvant therapy, patients undergoing BCS demonstrated significantly improved long-term survival outcomes compared with those treated with mastectomy, including higher 10-year overall survival and disease-free survival rates [24]. However, in our study, although the estimated median OS was numerically longer in the mastectomy group, this difference was not statistically significant, and multivariable analyses demonstrated that the surgical approach had no independent effect on overall survival. Although the difference did not reach statistical significance, a numerically longer overall survival was observed in the mastectomy group. This finding should be interpreted with caution, as it may reflect baseline differences between patients undergoing mastectomy and those treated with breast-conserving surgery rather than a true effect of the surgical approach itself. In retrospective studies, surgical selection is often influenced by tumor characteristics, disease extent, and clinician judgment, which may introduce confounding factors that affect survival outcomes. This observation may therefore reflect potential selection bias related to differences in baseline tumor and patient characteristics.

In ILC, axillary management represents a distinct area of clinical challenge due to the frequent underestimation of nodal involvement at diagnosis and the limited rates of pathological response to neoadjuvant therapy. Analyses based on national databases have shown that, in selected ILC patients who demonstrate a clinical response after neoadjuvant systemic therapy, SLNB does not adversely affect overall survival when compared with ALND, suggesting that de-escalation of axillary surgery may be feasible [25]. In contrast, the lower rates of pathological complete response to neoadjuvant chemotherapy in ILC compared with IDC may lead to reduced treatment efficacy and potentially less favorable clinical outcomes, particularly in patient groups in whom the surgical approach is limited or delayed [26]. Taken together, these data suggest that in ILC, the effectiveness of surgical and axillary management is shaped not by the type of surgery alone, but rather by treatment response, nodal status, and biological characteristics specific to patient subgroups [25,26].

The main strengths of our study include a comprehensive survival analysis based on standardized single-center data and a median oncologic follow-up of 31.8 months in a relatively rare and biologically heterogeneous histological subtype such as ILC. The management of all patients at the same institution, according to similar surgical principles and contemporary pathological evaluation protocols, minimized the impact of potential confounding factors frequently encountered in multicenter studies, such as variations in surgical techniques and inter-pathologist variability. Given the infiltrative growth pattern of ILC, preoperative breast MRI was frequently used in our cohort to better evaluate tumor extent and detect multifocal or multicentric disease, which may assist in surgical decision-making. However, the retrospective design of the study inherently carries a risk of selection bias; unmeasured subjective factors such as tumor size, radiological extent of disease, patient age, and surgeon preference may have influenced survival outcomes in the determination of the surgical approach. As demonstrated in Table 4, patients undergoing mastectomy had more advanced baseline disease characteristics, including larger tumor size, higher nodal involvement, and increased rates of multifocal disease and neoadjuvant therapy, which may reflect baseline differences between groups rather than a true effect of the surgical approach. In addition, although the sample size was adequate for overall and subgroup analyses, the limited representation of pleomorphic and other rare ILC variants within the cohort restricts the ability to draw definitive conclusions for these subtypes. Another important limitation of this study is the relatively short follow-up duration. The median follow-up time of 31.8 months may be insufficient to fully capture the long-term clinical course of invasive lobular carcinoma, which is known for its tendency toward late recurrence. Therefore, the absence of a significant difference in survival outcomes between surgical groups at this follow-up interval should be interpreted with caution. Longer follow-up periods are needed to better evaluate potential differences in long-term oncological outcomes between breast-conserving surgery and mastectomy in patients with ILC. In addition, PFS was calculated from the date of diagnosis rather than the date of surgery. Although this approach allowed the use of a common starting point for all patients, including those receiving neoadjuvant therapy, variations in the interval between diagnosis and surgery may have introduced additional variability in survival estimates. Finally, the absence of molecular data, such as BRCA mutation status and genomic risk scores (e.g., Oncotype DX), in a substantial proportion of patients prevented a comprehensive evaluation of the relationship between surgical approach and genetic risk profile. Despite these limitations, our study provides clinically meaningful real-world evidence suggesting that breast-conserving surgery may be associated with oncological outcomes comparable to those of mastectomy in appropriately selected patients with invasive lobular carcinoma, although these findings should be interpreted with caution, given the relatively short follow-up period.

## 5. Conclusions

This study demonstrates that, in the surgical management of ILC, BCS appears to be associated with oncological outcomes comparable to those of mastectomy in terms of both overall and progression-free survival and may be safely applied in appropriately selected patients. Although the distinctive infiltrative growth pattern of ILC and the limitations of preoperative imaging modalities may complicate surgical margin management, our findings with a median follow-up of 31.8 months suggest that the type of surgery was not independently associated with survival outcomes.

ILC is characterized by a distinct biological behavior with a well-recognized risk of late recurrence, which underscores the importance of long-term follow-up in outcome studies. Therefore, the absence of a survival difference between surgical approaches in the present study should be interpreted with caution, and longer follow-up is needed to better define long-term oncological outcomes.

In clinical practice, the surgical decision-making process should not be based solely on histopathological subtype; tumor biology, radiological extent, and the feasibility of achieving negative surgical margins should be evaluated within a multidisciplinary framework. In this context, BCS may be considered an effective breast-preserving treatment option in appropriately selected patients with ILC, while acknowledging baseline differences between surgical groups.

## Figures and Tables

**Table 1:** Demographic and clinical characteristics of the patients.

		Min–Max			Median	Mean ± SD/*n*−%			
Age at diagnosis		28.0	–	85.0	53.0	53.8	±	11.7	
Age at diagnosis	<50					107		42.0%	
	≥50					148		58.0%	
Height (cm)		140.0	–	185.0	160.0	159.7	±	6.6	
Weight (kg)		42.0	–	145.0	70.0	71.2	±	13.3	
BMI (kg/m^2^)		18.7	–	40.4	27.6	27.9	±	4.7	
Menopausal status	Premenopausal					111		43.5%	
	Postmenopausal					144		56.5%	
Comorbidity	(−)					136		53.3%	
	(+)					119		46.7%	
ECOG PS	0					222		87.1%	
	I					29		11.4%	
	II					4		1.6%	
Family history	(−)					120		47.1%	
	Breast cancer					62		24.3%	
	Prostate cancer					3		1.2%	
	Ovarian cancer					2		0.8%	
	Other cancers					68		26.7%	
BRCA Status	BRCA−					30		11.8%	
	BRCA1+					4		1.6%	
	BRCA2+					4		1.6%	
	Unknow					217		85.1%	
Age at menopause		34.0	–	63.0	48.0	48.2	±	4.9	
Age at menarche		9.0	–	33.0	13.0	13.3	±	2.4	
Age at first childbirth		12.0	–	40.0	23.0	23.9	±	5.3	
Number of children		0.0	–	14.0	2.0	2.3	±	1.9	
Oral contraceptive use	(−)					245		96.1%	
	(+)					10		3.9%	
Duration of oral contraceptive use (months)		2.0	–	10.0	6.0	5.1	±	2.8	
Seconder malignancy	(−)					241		94.5%	
	(+)					14		5.5%	

**Table 2:** Histopathological and biological characteristics of the tumor.

		Min–Max			Median	Mean ± SD/*n* (%)		
Tumor size at diagnosis (mm)		5.0	–	95.0	19.0	22.2	±	14.4
ER%		0.0	–	100.0	90.0	85.2	±	20.5
PR%		0.0	–	100.0	80.0	63.9	±	31.0
Ki-67		0.0	–	75.0	14.5	15.6	±	10.2
Ki-67	<%20					151		59.2%
	≥%20					87		34.1%
	Unknown					17		6.7%
ER%	(−)					22		8.6%
	(+)					233		91.4%
PR%	(−)					46		18%
	(+)					209		81.9%
HER2	(−)					238		93.3%
	(+)					17		6.6%
Histological subtype	Classic					196		76.9%
	Alveolar					27		10.6%
	Pleomorphic					12		4.7%
	Solid					8		3.1%
	Tubulolobular					3		1.2%
Lymphovascular invasion	(−)					177		69.4%
	(+)					78		30.6%
Multicentricity	(−)					202		79.2%
	(+)					53		20.8%
Grade	I					23		8.1%
	II					122		47.8%
	III					20		7.8%
	Not available					90		35.3%

**Table 3:** Treatment characteristics.

		Min–Max			Median	Mean ± SD/*n* (%)		
Axillary biopsy	Negative					96		37.6%
	Positive					66		25.9%
	Not performed					93		36.5%
Neoadjuvant Therapy	(−)					193		75.7%
	(+)					62		24.3%
Surgery	Not performed					20		7.8%
	Performed					235		92.2%
Type of Surgery	BCS					94		40.0%
	Mastectomy					141		60.0%
Type of axillary surgery	None					22		8.6%
	Limited axillary surgery					193		75.7%
	ALND/axillary curettage					40		15.7%
Stage	I A					56		21.9%
	II A					76		29.8%
	II B					49		19.2%
	III A					28		11.0%
	III B					13		5.0%
	III C					11		4.3%
	IV					22		8.6%
Host defense factor	(−)					212		83.1%
	(+)					43		16.9%
Necrosis	(−)					250		98.0%
	(+)					5		2.0%
Surgical margin	Negative					206		87.6%
	Positive					29		12.4%
***In patients receiving neoadjuvant treatment***								
Residual tumor burden (RBC-3)		0.0	–	4.7	0.0	1.3	±	1.9
Regression fibrosis rate (%)		0.0	–	100.0	30.0	35.5	±	33.1
E-cadherin loss	Negative					45		17.6%
	Positive					67		26.3%
	Unknown					143		56.1%
First-line chemotherapy	(−)					150		58.8
	(+)					98		38.4%
	Other treatments					7		2.7%
Radiotherapy	(−)					67		26.3%
	Adjuvant RT					170		66.7%
	Palliative RT					18		7.1%
Adjuvant endocrine therapy	None					22		8.6%
	Aİ					135		52.9%
	TMX					41		16.0%
	LHRH + TMX					35		13.7%
	LHRH + Aİ					26		10.2%
Progression/recurrence/metastasis	(−)					221		86.7%
	(+)					34		13.3%
Death	(−)					232		91.0%
	(+)					23		9.0%

**Table 4:** Baseline demographic, clinical, and treatment characteristics stratified by surgical approach.

Variable	BCS (*n* = 94)	Mastectomy (*n* = 141)	*p* Value
Age (years), median (range)	56 (36–84)	49 (28–85)	**0.002** ^a^
<50 years, *n* (%)	30 (31.9%)	72 (51.1%)	**0.006** ^b^
≥50 years, *n* (%)	64 (68.1%)	69 (48.9%)	
Menopausal status, *n* (%)			
Premenopausal	34 (36.2%)	74 (52.5%)	**0.020** ^b^
Postmenopausal	60 (63.8%)	67 (47.5%)	
BMI (kg/m^2^), median (range) ‡	29.4 (19.8–39.0)	27.1 (18.7–39.5)	**0.008** ^a^
Comorbidity, *n* (%)	52 (55.3%)	58 (41.1%)	**0.045** ^b^
Family history of cancer, *n* (%)	51 (54.3%)	77 (54.6%)	1.000 ^b^
Tumor size (mm), median (range)	22 (2–80)	29 (0–150)	**<0.001** ^a^
Clinical T stage, *n* (%)			
T1	45 (47.9%)	53 (37.6%)	0.185 ^b^
T2	43 (45.7%)	71 (50.4%)	
T3	3 (3.2%)	7 (5.0%)	
T4	2 (2.1%)	10 (7.1%)	
Clinical N stage, *n* (%)			
N0	79 (84.0%)	92 (65.2%)	**0.002** ^b^
N1	14 (14.9%)	45 (31.9%)	
N2	1 (1.1%)	3 (2.1%)	
N3	0 (0.0%)	1 (0.7%)	
Pathologic stage, *n* (%)			
Stage IA	28 (29.8%)	28 (19.9%)	**0.004** ^b,†^
Stage IIA	32 (34.0%)	44 (31.2%)	
Stage IIB	24 (25.5%)	25 (17.7%)	
Stage IIIA	7 (7.4%)	21 (14.9%)	
Stage IIIB/IIIC	2 (2.1%)	20 (14.2%)	
Histologic grade, *n* (%) §			
Grade 1	3 (3.6%)	2 (2.2%)	0.528 ^b^
Grade 2	68 (81.9%)	80 (87.9%)	
Grade 3	11 (13.3%)	8 (8.8%)	
Estrogen receptor (ER), *n* (%)			
Positive (≥1%)	93 (98.9%)	136 (96.5%)	0.406 ^c^
Negative	1 (1.1%)	5 (3.5%)	
Progesterone receptor (PR), *n* (%)			
Positive (≥1%)	80 (85.1%)	119 (84.4%)	1.000 ^b^
Negative	14 (14.9%)	22 (15.6%)	
HER2 status, *n* (%)			
Positive	6 (6.4%)	7 (5.0%)	0.872 ^b^
Negative	88 (93.6%)	133 (95.0%)	
Ki-67, *n* (%) ¶			
<20%	58 (62.4%)	91 (65.0%)	0.787 ^b^
≥20%	35 (37.6%)	49 (35.0%)	
Multicentric tumor, *n* (%)	13 (13.8%)	39 (27.7%)	**0.019** ^b^
Neoadjuvant chemotherapy, *n* (%)	9 (9.6%)	50 (35.5%)	**<0.001** ^b^
Adjuvant chemotherapy, *n* (%)	35 (37.2%)	53 (37.6%)	1.000 ^b^
Adjuvant endocrine therapy, *n* (%)			
None	4 (4.3%)	9 (6.4%)	**0.005** ^b^
Tamoxifen	19 (20.2%)	20 (14.2%)	
Aromatase inhibitor (AI)	58 (61.7%)	63 (44.7%)	
LHRH agonist + AI	3 (3.2%)	20 (14.2%)	
LHRH agonist + Tamoxifen	9 (9.6%)	26 (18.4%)	
Adjuvant radiotherapy, *n* (%)	88 (93.6%)	81 (57.4%)	**<0.001** ^b^

^a^ Mann–Whitney U test (continuous variables). ^b^ Chi-square test (categorical variables). ^c^ Fisher’s exact test (used when expected cell count < 5). ^†^ Stage IB excluded (*n* = 0 in both groups); Stages IIIB and IIIC merged owing to small cell counts. ‡ BMI available for 61 BCS and 108 mastectomy patients. § Histologic grade available for 83 BCS and 91 mastectomy patients. ¶ Ki-67 available for 93 BCS and 140 mastectomy patients. Bold *p* values indicate statistical significance (*p* < 0.05).

**Table 5:** Progression-free survival (PFS) outcomes according to surgical approach.

		Progression-Free Survival (Months)	95% CI			*p*
Surgical type	BCS	87.4	82.4	–	92.3	0.135
	Mastectomy	86.7	79.6	–	93.9	
Total		86.3	80.6	–	92.0	
Kaplan–Meier analysis (log-rank test)						

**Table 6:** Overall survival (OS) outcomes according to surgical approach.

		Overall Survival (Months)	95% CI			*p*
Surgical type	BCS	87.7	83.1	–	92.3	
	Mastectomy	115.7	106.8	–	124.7	
Total		112.3	105.0	–	119.6	
Kaplan–Meier analysis (log-rank test)						

**Table 7:** Effect of surgical type on overall survival in subgroup analyses: Cox proportional hazards regression.

Subgroup	BCS (*n* = 94)	Mastectomy (*n* = 141)	Events	HR	95% CI	*p*
Age						
<50 years	30	72	3	—	—	0.331 †
≥50 years	64	69	11	1.296	0.378–4.443	0.680
Tumor size						
≤20 mm	43	44	2	1.367	0.085–22.084	0.826
>20 mm	51	97	12	1.305	0.352–4.833	0.690
Pathologic stage						
Stage I	28	28	0	—	—	— ‡
Stage II	56	69	3	1.263	0.114–13.967	0.849
Stage III	9	41	10	0.573	0.147–2.231	0.422
Nodal involvement						
Negative (N0)	79	92	6	1.512	0.276–8.281	0.634
Positive (N1–N3)	15	49	8	1.021	0.205–5.091	0.979
Multifocal/multicentric tumor						
Absent	79	102	13	1.595	0.490–5.186	0.438
Present	13	39	1	—	—	0.375 †
Neoadjuvant chemotherapy						
No	85	91	6	0.890	0.179–4.424	0.887
Yes	9	50	8	1.098	0.134–9.023	0.931
Adjuvant chemotherapy						
No	57	87	8	4.895	0.600–39.920	0.138
Yes	35	53	5	0.798	0.133–4.797	0.805
Ki-67						
<20%	58	91	11	1.135	0.331–3.886	0.841
≥20%	35	49	3	—	—	0.375 †
Surgical margin						
Negative	78	128	11	1.614	0.428–6.090	0.480
Positive	16	13	3	1.837	0.163–20.726	0.623

HR > 1 indicates a higher hazard in the mastectomy group relative to BCS. † HR could not be estimated owing to complete separation (all events occurred exclusively in the mastectomy group with no events in the BCS group); *p* value derived from the log-rank test. ‡ Analysis not performed owing to zero events in both surgical groups within this subgroup. Bold *p* values indicate statistical significance (*p* < 0.05).

## Data Availability

The datasets used and/or analyzed during the current study are available from the corresponding author upon reasonable request.
